# Supplementary material for: Plastic multicellular development of Myxococcus xanthus: genotype–environment interactions in a physical gradient
Source: R Soc Open Sci. 2019 Mar 20;6(3):181730. doi: 10.1098/rsos.181730 (PMC6458408; doi:10.1098/rsos.181730)
Supplement: Extended experimental and analytical results [file rsos181730supp1.docx]

**Plastic multicellular development of *Myxococcus xanthus*: genotype-environment interactions in a physical gradient**

***Natsuko Rivera-Yoshida^1,2,3^, Alejandro V. Arzola^4^, Juan A. Arias Del Angel^1,2,3^, Alessio Franci^5^, Michael Travisano^6^, Ana E. Escalante^1^*, Mariana Benítez^1,2^****

1 Laboratorio Nacional de Ciencias de la Sostenibilidad (LANCIS), Instituto de Ecología, Universidad Nacional Autónoma de México, Mexico City, Mexico

2 Centro de Ciencias de la Complejidad, Universidad Nacional Autónoma de México, Mexico City, Mexico

3 Programa de Doctorado en Ciencias Biomédicas, Universidad Nacional Autónoma de México, Mexico

4 Instituto de Física, Universidad Nacional Autónoma de México, Apdo. Postal 20-364, 01000 Cd. de México, Mexico

5 Universidad Nacional Autonóma de México, Facultad de Ciencias, Mexico

6 Department of Ecology, Evolution and Behavior, University of Minnesota, Saint Paul, MN, USA.

* [mbenitez@iecologia.unam.mx](mailto:mbenitez@iecologia.unam.mx), aescalante@iecologia.unam.mx

**Supplementary Information**


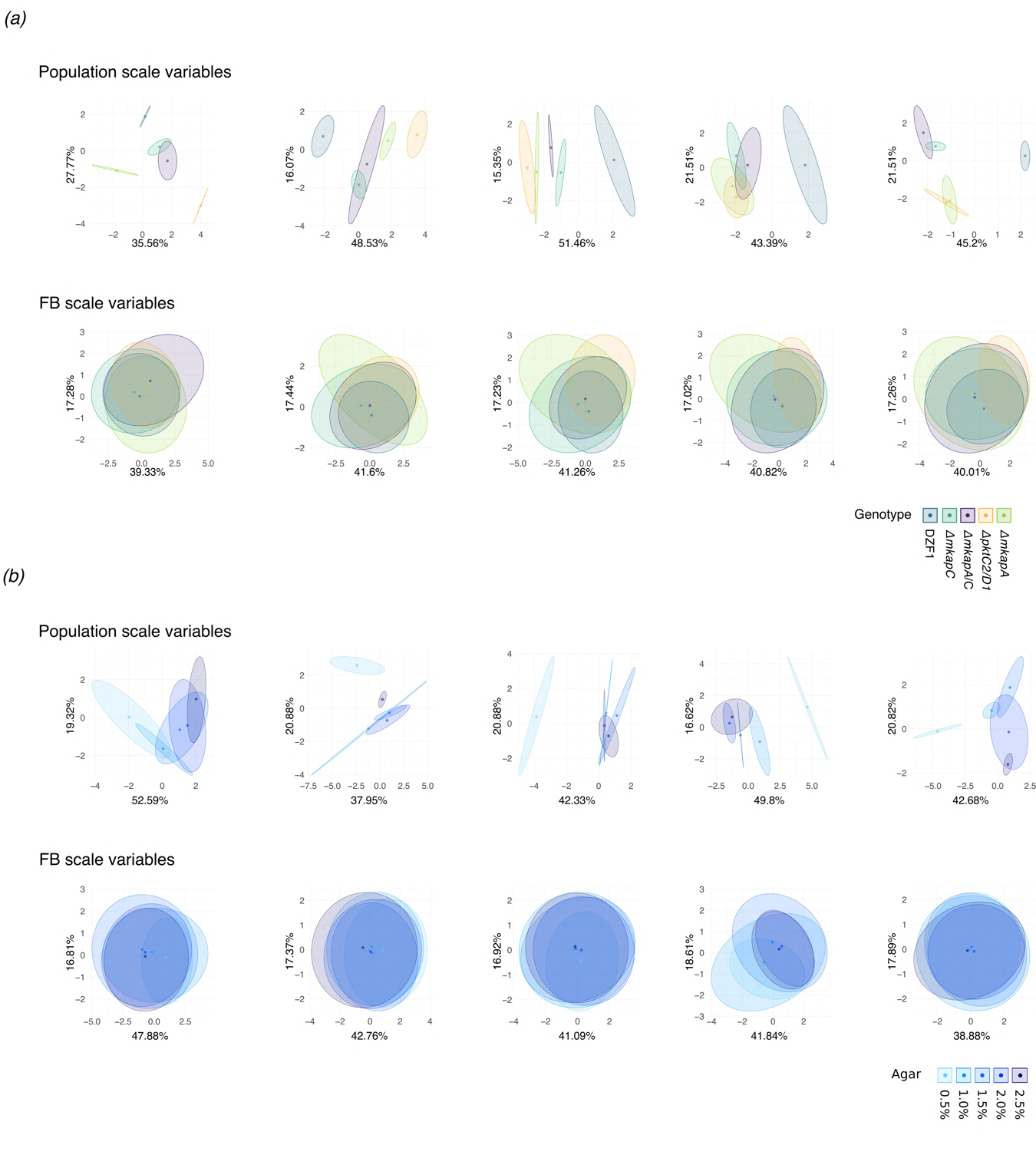


**Figure S1.** FAMD analysis based on data grouped by phenotypic expression scale (FB scale, population scale) per *(a)* substrate agar concentration and *(b)* genotype. 95% confidence interval ellipses enclose data centroids.


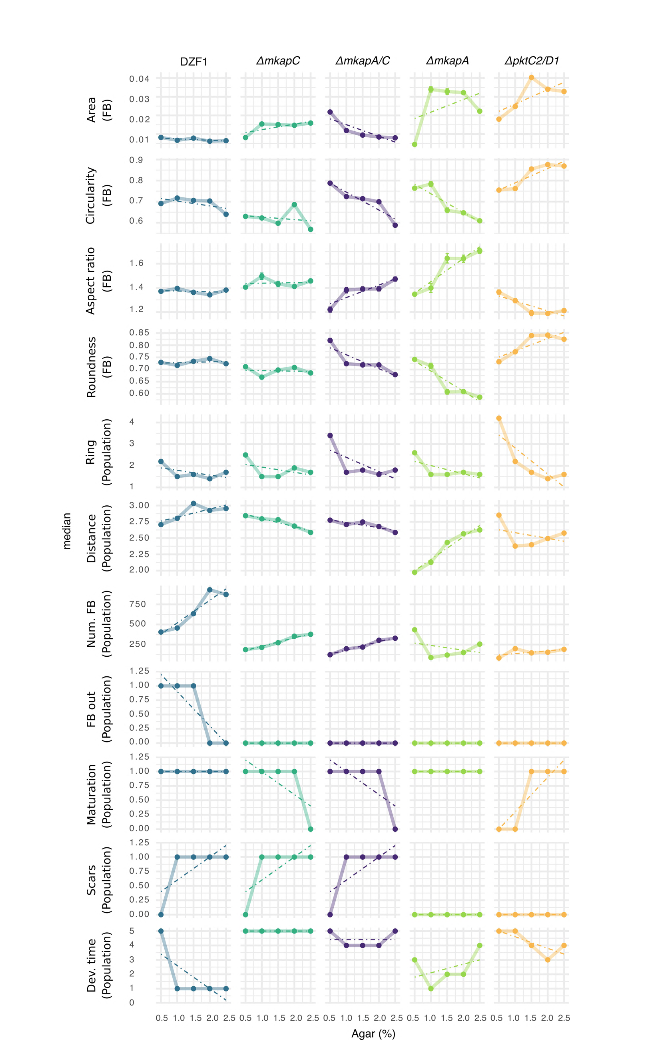


**Figure S2.** Reaction norms of all considered phenotypic traits among *Myxococcus xanthus* parental and mutant genotypes in response to substrate agar concentration. Each line represents the reaction norm of a single genotype (columns) based on the median ± standard error of single phenotypic traits (rows). Linear regression fit with a dotted line, although p-values are not significant in all cases.

| **Phenotypic trait (short name)** | **Phenotypic trait** | **Formula / Symbol** | **Phenotypic scale** |
| --- | --- | --- | --- |
| Area | Area | mm² | FB |
| Circularity | Circularity  (Shape descriptor) | 4π * [Area]/[Perimeter]²  Ranges from  0 (infinitely elongated polygon) to  1 (perfect circle)  Dimensionless | FB |
| Aspect ratio | Aspect ratio  (Shape descriptor) | [Major axis] / [Minor axis]  Dimensionless | FB |
| Roundness | Roundness  (Shape descriptor) | 4 * [Area] / π × [Major axis]  Dimensionless | FB |
| Ring | Ring formation at the  edge of the drop | max[g(r)] / mean[g(r)]  Where g is the density of gray values, in the inverted image, as a function of the radial position r.  mm⁻² | Population |
| Distance | Standard distance  between FBs | √ [Σ((coordsX - mcX)² + (coordsY - mcY)²)] /  Num. FB  Where  coordsX, coordsY = FB coordinates on X and Y axis, respectively  mcX, mcY = mean center coordinate on X and Y axis respectively  mm | Population |
| Num. FB | Number of FB | Count of FB | Population |
| FB out | FB outside the edge of the drop | 0 (No)  1 (Yes) | Population |
| Maturation | Complete maturation (fully developed/complete darkening) of FB at 96h | 0 (No)  1 (Yes) | Population |
| Scars | Vestiges of not clearly defined FB | 0 (No)  1 (Yes) | Population |
| Dev. time | Developmental time (full development from 0h to 96h) | Range from  1 (fast development) 5 (slow development) | Population |

**Table S1.** Phenotypic traits, formulas and scales.

| **Strain: DZF1** | **Dim.1 (33.97%)** | **Dim.2 (19.46%)** | **Dim.3 (11.77%)** | **Dim.4 (8.99%)** | **Dim.5 (8.46%)** |
| --- | --- | --- | --- | --- | --- |
| **Area** | 0.36 | 0.03 | 2.42 | 69.13 | 1.59 |
| **Circularity** | 0.00 | **22.16** | 0.40 | 3.22 | 0.00 |
| **Aspect ratio** | 0.00 | **38.62** | 0.42 | 1.09 | 0.03 |
| **Roundness** | 0.01 | **38.41** | 0.40 | 1.18 | 0.10 |
| **Ring** | **18.43** | 0.03 | 9.76 | 0.09 | 0.71 |
| **Distance** | 15.64 | 0.00 | 0.46 | 1.75 | 1.82 |
| **Number FB** | 14.69 | 0.01 | 10.73 | 1.95 | 1.20 |
| **FB out the drop** | 6.30 | 0.15 | 43.28 | 0.14 | 0.59 |
| **Complete Maturation** | 0.00 | 0.00 | 0.00 | 0.00 | 0.00 |
| **Scars** | **22.24** | 0.02 | 2.12 | 0.19 | 0.72 |
| **Dev. time** | **22.24** | 0.02 | 2.12 | 0.19 | 0.72 |
| **Replica** | 0.09 | 0.55 | 27.89 | 21.06 | 92.53 |

| **Strain: *ΔmkapC*** | **Dim.1 (26.40%)** | **Dim.2 (22.32%)** | **Dim.3 (13.70%)** | **Dim.4 (9.33%)** | **Dim.5 (9.02%)** |
| --- | --- | --- | --- | --- | --- |
| **Area** | 1.98 | 1.96 | 5.73 | 4.38 | 1.58 |
| **Circularity** | 3.11 | **22.33** | 1.03 | 0.11 | 0.02 |
| **Aspect ratio** | 2.31 | **33.42** | 0.02 | 0.00 | 0.03 |
| **Roundness** | 2.13 | **34.20** | 0.31 | 0.02 | 0.01 |
| **Ring** | 11.93 | 0.00 | 35.57 | 2.07 | 0.30 |
| **Distance** | **18.34** | 3.21 | 15.64 | 0.77 | 0.00 |
| **Number FB** | **21.99** | 2.96 | 4.64 | 1.83 | 0.03 |
| **FB out the drop** | 0.00 | 0.00 | 0.00 | 0.00 | 0.00 |
| **Complete Maturation** | 17.17 | 1.23 | 12.09 | 3.38 | 1.01 |
| **Scars** | **20.26** | 0.46 | 21.50 | 0.03 | 0.76 |
| **Dev. time** | 0.00 | 0.00 | 0.00 | 0.00 | 0.00 |
| **Replica** | 0.78 | 0.24 | 3.46 | 87.41 | 96.26 |

| **Strain: *ΔmkapA/ΔmkapC*** | **Dim.1 (27.01%)** | **Dim.2 (18.57%)** | **Dim.3 (15.97%)** | **Dim.4 (10.43%)** | **Dim.5 (9.92%)** |
| --- | --- | --- | --- | --- | --- |
| **Area** | 2.28 | 2.03 | 2.85 | 0.03 | 6.88 |
| **Circularity** | 13.40 | 9.06 | 0.98 | 0.11 | 0.59 |
| **Aspect ratio** | 12.68 | 11.32 | 11.90 | 0.60 | 0.02 |
| **Roundness** | **13.81** | 10.15 | 11.75 | 0.48 | 0.06 |
| **Ring** | 11.15 | **19.07** | 1.60 | 6.88 | 2.85 |
| **Distance** | 6.15 | 0.97 | 11.82 | 19.59 | 3.08 |
| **Number FB** | **16.48** | 3.83 | 9.97 | 0.02 | 8.33 |
| **FB out the drop** | 0.00 | 0.00 | 0.00 | 0.00 | 0.00 |
| **Complete Maturation** | 7.68 | 4.29 | 29.14 | 4.35 | 0.46 |
| **Scars** | **15.85** | **17.57** | 0.03 | 1.43 | 1.17 |
| **Dev. time** | 0.05 | **21.24** | 18.08 | 10.05 | 0.25 |
| **Replica** | 0.48 | 0.46 | 1.87 | 56.46 | 76.29 |

| **Strain: *ΔmkapA*** | **Dim.1 (38.71%)** | **Dim.2 (16.42%)** | **Dim.3 (11.46%)** | **Dim.4 (10.01%)** | **Dim.5 (7.12%)** |
| --- | --- | --- | --- | --- | --- |
| **Area** | 14.82 | 3.29 | 0.50 | 5.10 | 1.42 |
| **Circularity** | **15.21** | 8.35 | 0.92 | 0.47 | 0.03 |
| **Aspect ratio** | **14.86** | 15.29 | 3.94 | 1.64 | 0.43 |
| **Roundness** | 14.84 | **15.50** | 3.57 | 0.74 | 0.61 |
| **Ring** | **16.17** | 11.32 | 1.82 | 9.75 | 0.30 |
| **Distance** | 11.28 | 0.62 | 7.66 | 35.77 | 0.41 |
| **Number FB** | 12.03 | **17.65** | 5.36 | 6.98 | 0.00 |
| **FB out the drop** | 0.00 | 0.00 | 0.00 | 0.00 | 0.00 |
| **Complete Maturation** | 0.00 | 0.00 | 0.00 | 0.00 | 0.00 |
| **Scars** | 0.00 | 0.00 | 0.00 | 0.00 | 0.00 |
| **Dev. time** | 0.52 | **26.22** | 28.43 | 9.50 | 0.00 |
| **Replica** | 0.27 | 1.76 | 47.80 | 30.05 | 96.81 |

| **Strain: *ΔpktC2/ΔpktD1*** | **Dim.1 (30.54%)** | **Dim.2 (20.81%)** | **Dim.3 (15.06%)** | **Dim.4 (9.69%)** | **Dim.5 (9.11%)** |
| --- | --- | --- | --- | --- | --- |
| **Area** | 3.85 | 8.17 | 9.86 | 1.16 | 1.02 |
| **Circularity** | 15.24 | 8.37 | 0.05 | 0.57 | 0.16 |
| **Aspect ratio** | 10.38 | **18.87** | 8.65 | 0.34 | 0.04 |
| **Roundness** | 11.52 | **16.94** | 9.36 | 0.52 | 0.03 |
| **Ring** | **17.72** | 13.90 | 1.74 | 0.20 | 0.46 |
| **Distance** | 2.48 | **18.13** | 14.20 | 7.87 | 1.06 |
| **Number FB** | 0.84 | 12.80 | 29.77 | 1.60 | 0.00 |
| **FB out the drop** | 0.00 | 0.00 | 0.00 | 0.00 | 0.00 |
| **Complete Maturation** | **19.06** | 1.30 | 12.21 | 2.37 | 0.11 |
| **Scars** | 0.00 | 0.00 | 0.00 | 0.00 | 0.00 |
| **Dev. time** | **18.68** | 0.56 | 12.26 | 0.35 | 1.79 |
| **Replica** | 0.23 | 0.97 | 1.90 | 85.03 | 95.32 |

| **Agar: 0.5%** | **Dim.1 (25.19%)** | **Dim.2 (21.39%)** | **Dim.3 (17.53%)** | **Dim.4 (8.50%)** | **Dim.5 (8.23%)** |
| --- | --- | --- | --- | --- | --- |
| **Area** | 6.50 | 2.18 | 0.07 | 1.08 | 2.39 |
| **Circularity** | 1.66 | **19.47** | 2.86 | 0.41 | 0.17 |
| **Aspect ratio** | 0.04 | **17.41** | 22.81 | 0.00 | 0.06 |
| **Roundness** | 0.02 | **17.91** | 22.10 | 0.00 | 0.01 |
| **Ring** | 8.04 | 14.78 | 14.06 | 0.61 | 0.01 |
| **Distance** | **20.44** | 3.63 | 5.40 | 0.35 | 3.58 |
| **Number FB** | **26.53** | 0.99 | 0.35 | 0.32 | 1.82 |
| **FB out the drop** | 0.14 | 13.26 | 18.58 | 0.38 | 2.85 |
| **Complete Maturation** | 11.39 | 5.78 | 5.73 | 0.04 | 4.05 |
| **Dev. time** | **22.53** | 4.33 | 7.15 | 0.81 | 0.08 |
| **Replica** | 2.70 | 0.26 | 0.89 | 96.01 | 85.00 |

| **Agar: 1.0%** | **Dim.1 (35.71%)** | **Dim.2 (19.91%)** | **Dim.3 (10.73%)** | **Dim.4 (9.56%)** | **Dim.5 (7.81%)** |
| --- | --- | --- | --- | --- | --- |
| **Area** | 6.82 | 0.12 | 2.07 | 17.66 | 2.64 |
| **Circularity** | 0.16 | **25.90** | 0.28 | 0.00 | 0.08 |
| **Aspect ratio** | 0.03 | **33.13** | 2.55 | 0.84 | 0.10 |
| **Roundness** | 0.13 | **32.72** | 3.08 | 0.87 | 0.03 |
| **Ring** | 13.86 | 0.45 | 13.28 | 5.10 | 0.07 |
| **Distance** | 12.41 | 0.40 | 6.88 | 12.84 | 0.90 |
| **Number FB** | **13.94** | 1.98 | 12.28 | 0.00 | 0.91 |
| **FB out the drop** | **14.88** | 1.88 | 11.40 | 1.56 | 0.97 |
| **Complete Maturation** | 12.38 | 0.85 | 5.72 | 1.21 | 2.28 |
| **Scars** | **15.32** | 0.48 | 5.36 | 8.52 | 0.01 |
| **Dev. time** | 9.69 | 1.61 | 13.36 | 20.04 | 5.32 |
| **Replica** | 0.37 | 0.48 | 23.74 | 31.37 | 86.70 |

| **Agar: 1.5%** | **Dim.1 (37.74%)** | **Dim.2 (20.44%)** | **Dim.3 (11.61%)** | **Dim.4 (8.96%)** | **Dim.5 (8.19%)** |
| --- | --- | --- | --- | --- | --- |
| **Area** | 9.31 | 0.16 | 9.20 | 8.86 | 0.11 |
| **Circularity** | 0.13 | **27.59** | 0.05 | 0.48 | 0.21 |
| **Aspect ratio** | 1.24 | **34.02** | 0.00 | 1.44 | 0.07 |
| **Roundness** | 0.77 | **34.77** | 0.02 | 1.30 | 0.03 |
| **Ring** | 6.11 | 1.32 | 25.46 | 4.70 | 0.01 |
| **Distance** | **19.25** | 0.53 | 3.27 | 1.17 | 0.00 |
| **Number FB** | **20.96** | 0.10 | 0.32 | 1.74 | 0.11 |
| **FB out the drop** | **19.06** | 0.03 | 0.45 | 8.84 | 1.07 |
| **Complete Maturation** | 0.00 | 0.00 | 0.00 | 0.00 | 0.00 |
| **Dev. time** | 12.45 | 0.01 | 2.70 | 28.11 | 2.82 |
| **Replica** | 0.67 | 0.12 | 45.53 | 22.37 | 94.79 |

| **Agar: 2.0%** | **Dim.1 (32.87%)** | **Dim.2 (20.27%)** | **Dim.3 (14.37%)** | **Dim.4 (10.41%)** | **Dim.5 (6.41%)** |
| --- | --- | --- | --- | --- | --- |
| **Area** | 11.01 | 1.76 | 4.21 | 4.69 | 1.25 |
| **Circularity** | 0.71 | **25.92** | 0.41 | 0.00 | 0.17 |
| **Aspect ratio** | 2.97 | **29.21** | 2.94 | 1.64 | 0.39 |
| **Roundness** | 2.24 | **30.56** | 2.68 | 1.38 | 0.46 |
| **Ring** | 9.64 | 1.78 | 13.52 | 15.37 | 0.00 |
| **Distance** | **19.78** | 3.28 | 4.38 | 0.77 | 0.07 |
| **Number FB** | **22.73** | 1.89 | 0.60 | 0.15 | 0.73 |
| **FB out the drop** | 6.63 | 0.01 | 23.56 | 13.07 | 0.01 |
| **Complete Maturation** | 0.00 | 0.00 | 0.00 | 0.00 | 0.00 |
| **Scars** | 6.98 | 5.31 | 9.78 | 16.07 | 10.56 |
| **Dev. time** | **16.79** | 0.04 | 2.99 | 8.86 | 13.50 |
| **Replica** | 0.54 | 0.22 | 34.94 | 38.00 | 72.84 |

| **Agar: 2.5%** | **Dim.1 (33.24%)** | **Dim.2 (19.83%)** | **Dim.3 (15.30%)** | **Dim.4 (8.48%)** | **Dim.5 (8.40%)** |
| --- | --- | --- | --- | --- | --- |
| **Area** | 7.31 | 4.68 | 5.28 | 0.24 | 1.70 |
| **Circularity** | 1.49 | **24.48** | 0.06 | 0.67 | 0.04 |
| **Aspect ratio** | 2.77 | **26.31** | 9.54 | 0.05 | 0.15 |
| **Roundness** | 2.36 | **27.92** | 8.66 | 0.08 | 0.06 |
| **Ring** | 3.74 | 1.38 | 20.06 | 1.94 | 0.00 |
| **Distance** | **22.45** | 1.77 | 0.00 | 0.14 | 0.90 |
| **Number FB** | **21.73** | 3.56 | 0.90 | 0.00 | 0.05 |
| **FB out the drop** | 0.00 | 0.00 | 0.00 | 0.00 | 0.00 |
| **Complete Maturation** | 11.70 | 1.35 | 23.17 | 1.04 | 0.12 |
| **Scars** | 3.21 | 8.13 | 30.48 | 0.79 | 0.05 |
| **Dev. time** | **23.15** | 0.37 | 1.07 | 0.00 | 0.01 |
| **Replica** | 0.08 | 0.05 | 0.79 | 95.05 | 96.93 |

**Table S2.** Dimension values of FAMD analysis for each phenotypic trait. Data was grouped by genotype and by agar concentration.


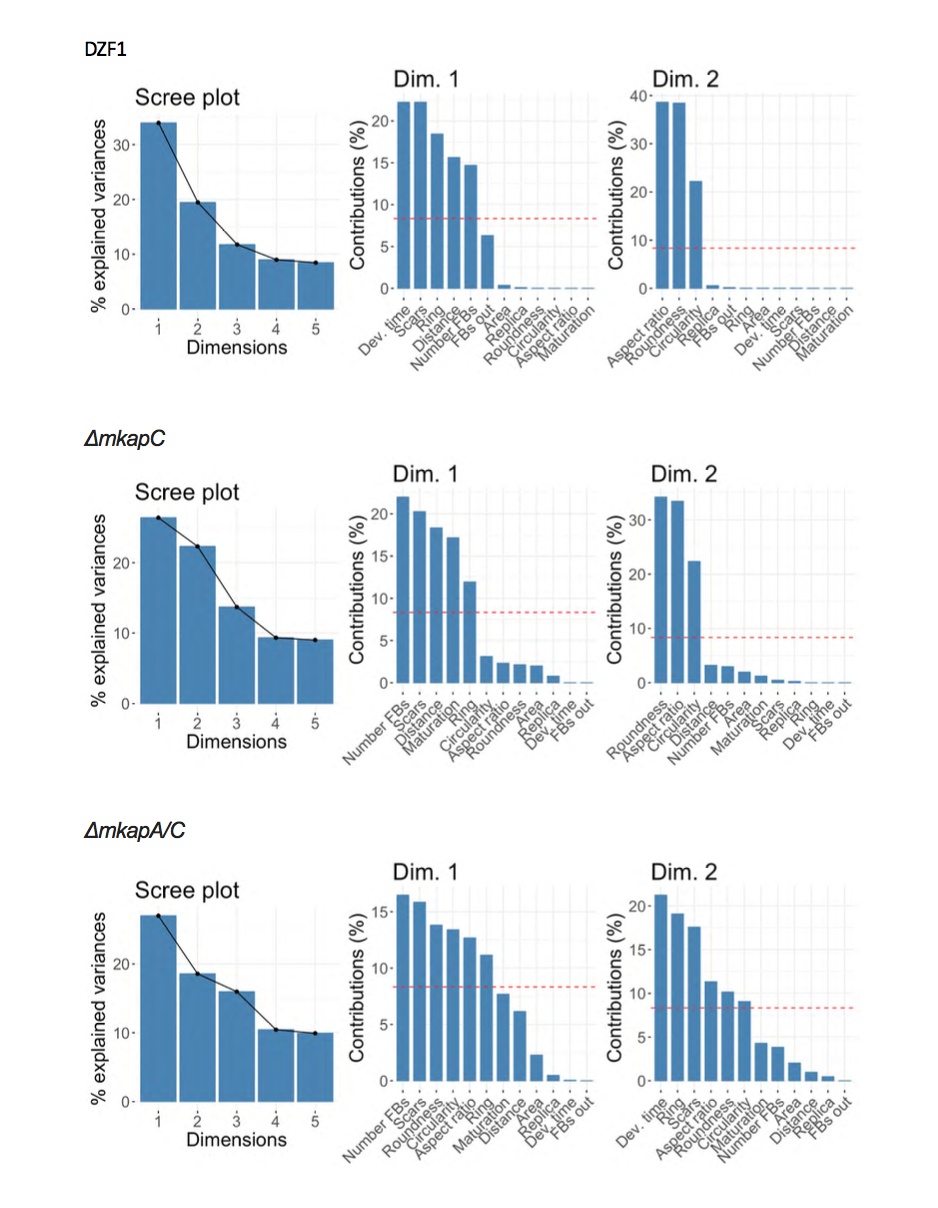


**
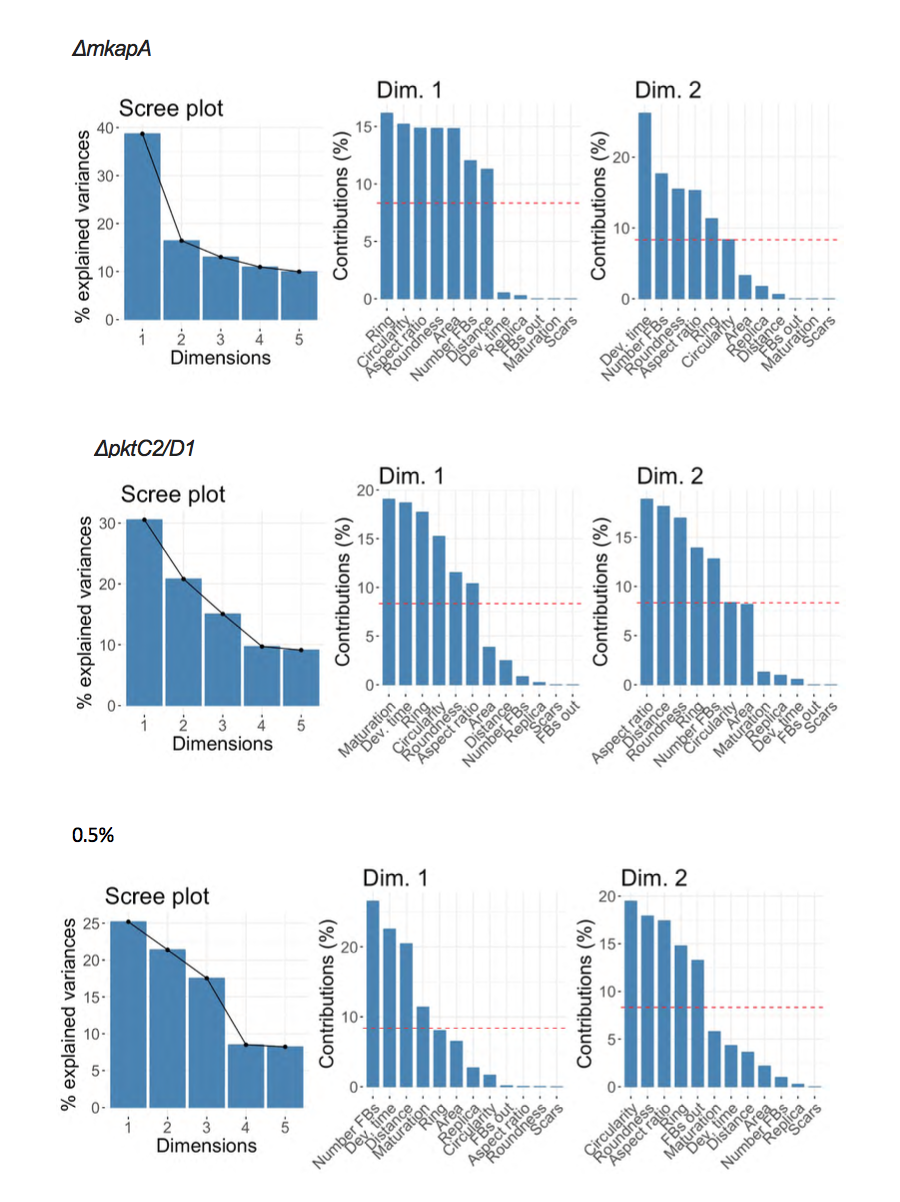
**


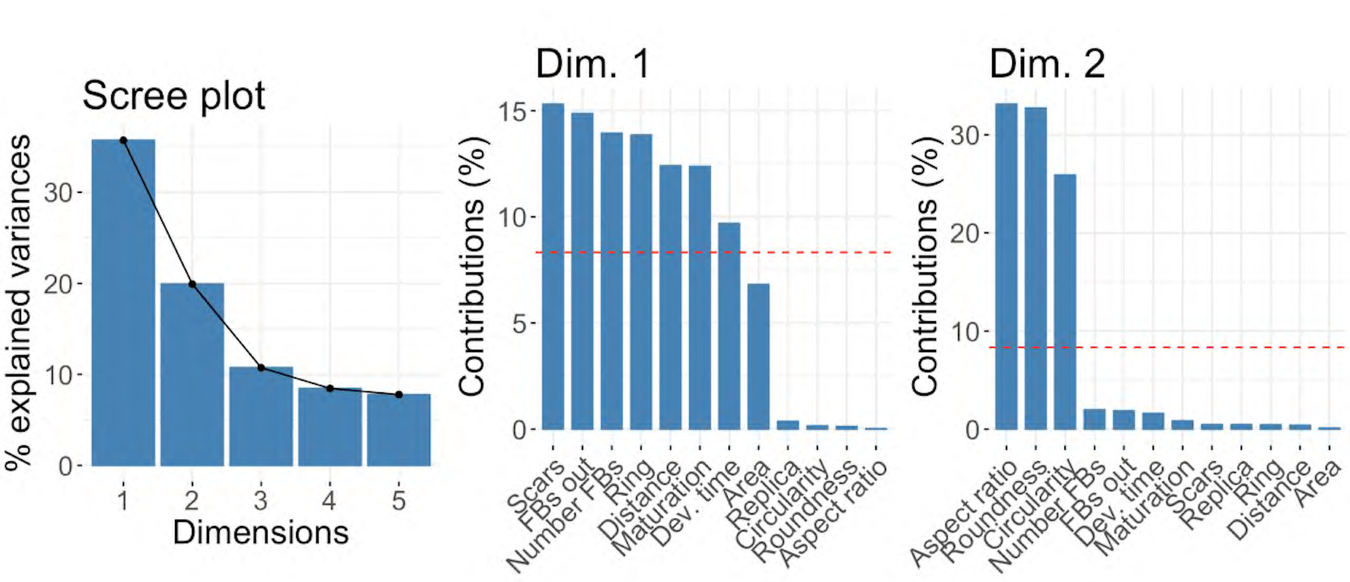

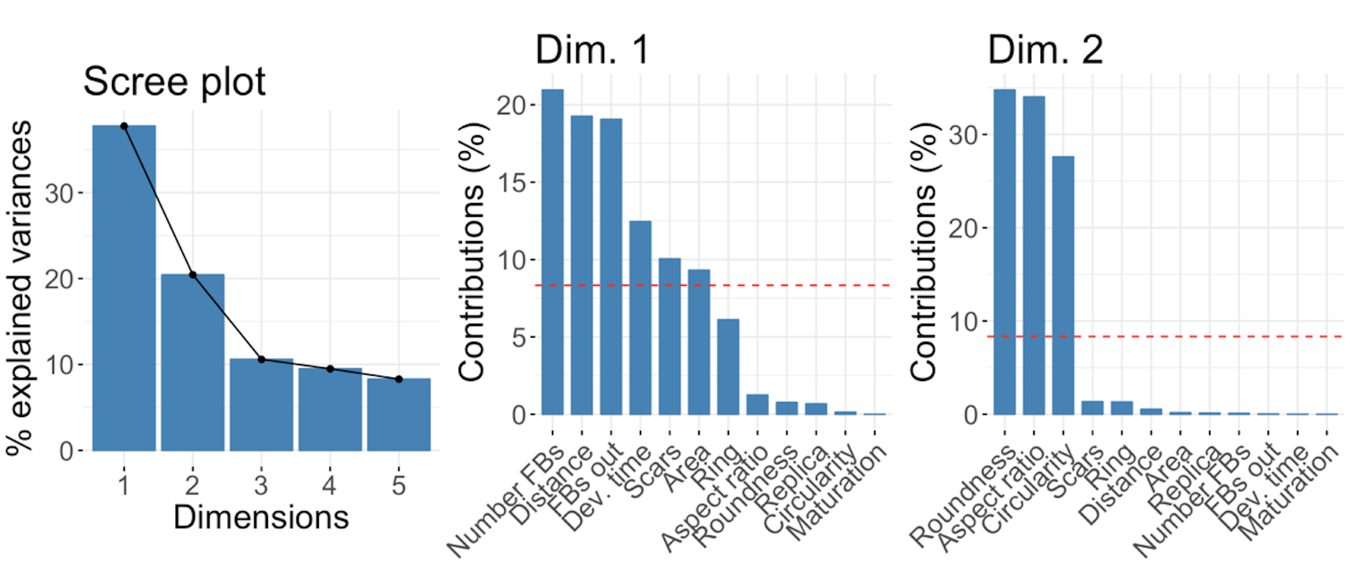

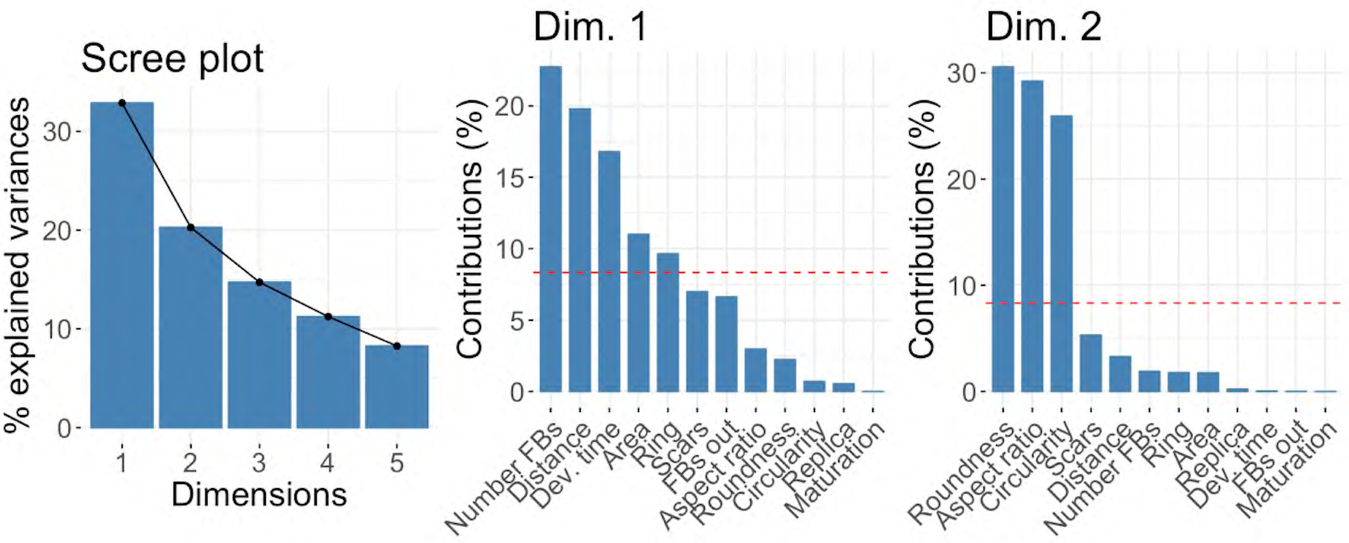


1.0%

1.5%

2.0%

2.5%


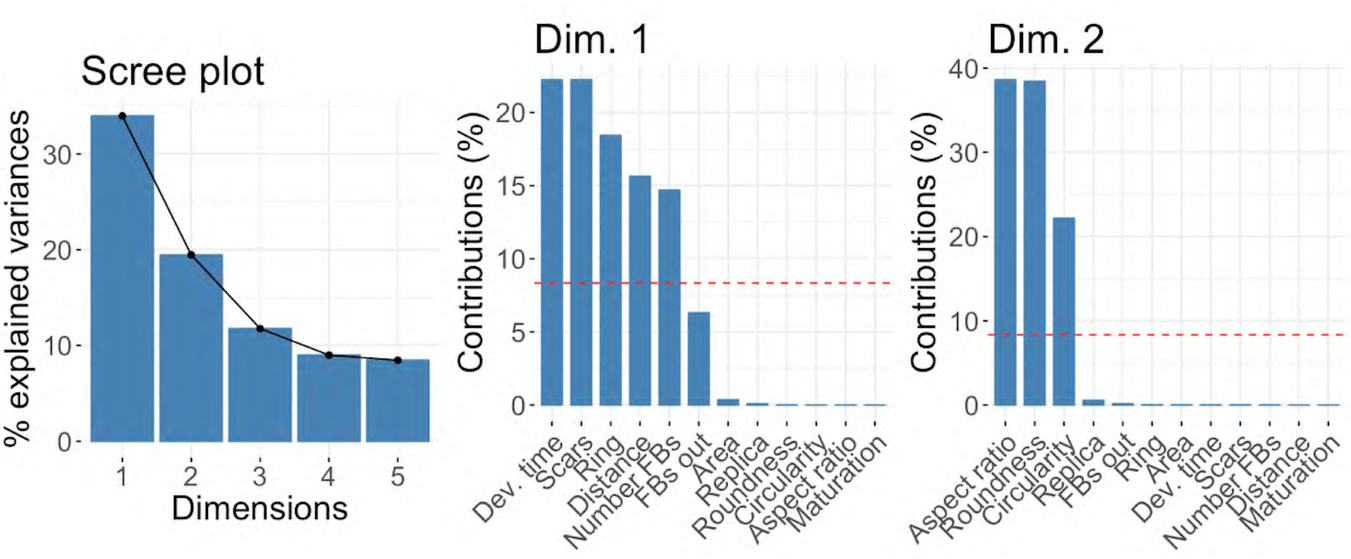


**Figure S3.** FAMD analysis summary. Data was grouped by genotype and by agar concentration. *(a)* Scree plot. Percentage of explained variances in the first five dimensions. *(b)* Contributions of variables to Dimension 1 and *(c)* contributions of variables to Dimension 2. The red dashed line indicates the expected average value.

*(a)*

| **Genotype/Strain** | **Trait** | **P value** | **F stat.** | **R2** | **Slope** | **Intercept** | **Sqr_err** |
| --- | --- | --- | --- | --- | --- | --- | --- |
| *ΔmkapA* | Area | 0.000 | 544.108 | 0.141 | 0.009 | 0.009 | 0.000 |
|  | Circularity | 0.000 | 679.817 | 0.170 | -0.080 | 0.785 | 0.022 |
|  | Aspect ratio | 0.000 | 417.361 | 0.112 | 0.224 | 1.356 | 0.284 |
|  | Roundness | 0.000 | 499.915 | 0.131 | -0.073 | 0.755 | 0.025 |
|  | Ring | 0.000 | 6839.785 | 0.673 | -0.540 | 2.841 | 0.101 |
|  | Distance | 0.000 | 15865.950 | 0.827 | 0.296 | 1.896 | 0.013 |
|  | Num. FB | 0.000 | 1387.754 | 0.295 | -110.439 | 471.363 | 20828.100 |
|  | FB out | NaN | NaN | NaN | 0.000 | 0.000 | 0.000 |
|  | Maturation | 0.307 | 3323.881 | 0.500 | 0.000 | 1.000 | 0.000 |
|  | Scars | NaN | NaN | NaN | 0.000 | 0.000 | 0.000 |
|  | Dev. time | 0.000 | 341.329 | 0.093 | 0.384 | 2.551 | 1.022 |
| *ΔmkapA/C* | Area | 0.000 | 13.073 | 0.004 | -0.002 | 0.021 | 0.000 |
|  | Circularity | 0.000 | 408.548 | 0.106 | -0.078 | 0.793 | 0.023 |
|  | Aspect ratio | 0.000 | 66.910 | 0.019 | 0.105 | 1.379 | 0.251 |
|  | Roundness | 0.000 | 101.390 | 0.029 | -0.042 | 0.764 | 0.027 |
|  | Ring | 0.000 | 1567.481 | 0.313 | -0.411 | 2.638 | 0.166 |
|  | Distance | 0.000 | 491.914 | 0.125 | -0.072 | 2.762 | 0.016 |
|  | Num. FB | 0.000 | 4615.038 | 0.573 | 94.328 | 106.963 | 2967.003 |
|  | FB out | NaN | NaN | NaN | 0.000 | 0.000 | 0.000 |
|  | Maturation | 0.000 | 4074.926 | 0.542 | -0.502 | 1.578 | 0.095 |
|  | Scars | 0.000 | 2307.834 | 0.401 | 0.290 | 0.391 | 0.056 |
|  | Dev. time | 0.000 | 463.223 | 0.119 | 0.295 | 3.836 | 0.289 |
| *ΔmkapC* | Area | 0.000 | 132.558 | 0.030 | 0.003 | 0.012 | 0.000 |
|  | Circularity | 0.000 | 25.115 | 0.006 | -0.018 | 0.635 | 0.027 |
|  | Aspect ratio | 0.280 | 1.165 | 0.000 | 0.014 | 1.589 | 0.321 |
|  | Roundness | 0.496 | 0.463 | 0.000 | -0.003 | 0.681 | 0.030 |
|  | Ring | 0.000 | 776.066 | 0.154 | -0.207 | 2.150 | 0.111 |
|  | Distance | 0.000 | 5663.661 | 0.571 | -0.129 | 2.927 | 0.006 |
|  | Num. FB | 0.000 | 35269.270 | 0.893 | 103.746 | 129.636 | 616.349 |
|  | FB out | NaN | NaN | NaN | 0.000 | 0.000 | 0.000 |
|  | Maturation | 0.000 | 4517.801 | 0.515 | -0.461 | 1.507 | 0.095 |
|  | Scars | 0.000 | 3816.291 | 0.473 | 0.345 | 0.281 | 0.063 |
|  | Dev. time | 0.087 | 4247.723 | 0.500 | 0.000 | 5.000 | 0.000 |
| *ΔpktC2/D1* | Area | 0.000 | 209.507 | 0.083 | 0.007 | 0.021 | 0.000 |
|  | Circularity | 0.000 | 461.348 | 0.166 | 0.075 | 0.675 | 0.013 |
|  | Aspect ratio | 0.000 | 74.236 | 0.031 | -0.096 | 1.506 | 0.129 |
|  | Roundness | 0.000 | 99.891 | 0.041 | 0.044 | 0.705 | 0.020 |
|  | Ring | 0.000 | 2337.550 | 0.503 | -0.877 | 3.409 | 0.344 |
|  | Distance | 0.002 | 9.170 | 0.004 | -0.013 | 2.518 | 0.018 |
|  | Num. FB | 0.000 | 202.778 | 0.081 | 16.852 | 140.398 | 1463.382 |
|  | FB out | NaN | NaN | NaN | 0.000 | 0.000 | 0.000 |
|  | Maturation | 0.000 | 6041.181 | 0.723 | 0.611 | -0.352 | 0.065 |
|  | Scars | NaN | NaN | NaN | 0.000 | 0.000 | 0.000 |
|  | Dev. time | 0.000 | 3920.039 | 0.629 | -0.946 | 5.610 | 0.239 |
| DZF1 | Area | 0.000 | 167.262 | 0.017 | -0.001 | 0.010 | 0.000 |
|  | Circularity | 0.000 | 74.269 | 0.007 | -0.016 | 0.704 | 0.015 |
|  | Aspect ratio | 0.907 | 0.014 | 0.000 | 0.001 | 1.446 | 0.115 |
|  | Roundness | 0.452 | 0.565 | 0.000 | 0.002 | 0.719 | 0.019 |
|  | Ring | 0.000 | 2448.239 | 0.197 | -0.161 | 1.897 | 0.047 |
|  | Distance | 0.000 | 4709.355 | 0.321 | 0.103 | 2.727 | 0.010 |
|  | Num. FB | 0.000 | 34552.620 | 0.776 | 277.660 | 259.570 | 9867.596 |
|  | FB out | 0.000 | 16259.910 | 0.620 | -0.588 | 1.552 | 0.094 |
|  | Maturation | 0.069 | 9947.936 | 0.500 | 0.000 | 1.000 | 0.000 |
|  | Scars | 0.000 | 8337.421 | 0.456 | 0.331 | 0.313 | 0.058 |
|  | Dev. time | 0.000 | 8337.421 | 0.456 | -1.323 | 3.750 | 0.929 |

*(b)*

| **Genotype/Strain** | **Trait** | **P value** | **F stat.** | **R2** | **Slope** | **Intercept** | **Sqr_err** |
| --- | --- | --- | --- | --- | --- | --- | --- |
| *ΔmkapA* | Dim. 1 FAMD | 0.001 | 18.187 | 0.583 | -2.468 | 3.702 | 2.176 |
| *ΔmkapA/C* |  | 0.000 | 54.204 | 0.659 | -2.698 | 4.047 | 1.880 |
| *ΔmkapC* |  | 0.000 | 61.712 | 0.589 | -2.493 | 3.740 | 2.165 |
| *ΔpktC2/D1* |  | 0.000 | 101.046 | 0.635 | -2.620 | 3.929 | 1.970 |
| DZF1 |  | 0.000 | 138.558 | 0.655 | -2.615 | 3.922 | 1.801 |
| *ΔmkapA* | Dim. 2 FAMD | 0.000 | 46.447 | 0.389 | -1.889 | 2.833 | 2.804 |
| *ΔmkapA/C* |  | 0.000 | 16.390 | 0.183 | -1.184 | 1.775 | 3.120 |
| *ΔmkapC* |  | 0.120 | 2.478 | 0.033 | -0.470 | 0.705 | 3.253 |
| *ΔpktC2/D1* |  | 0.653 | 0.204 | 0.003 | 0.120 | -0.181 | 2.600 |
| DZF1 |  | 0.015 | 6.179 | 0.078 | 0.603 | -0.905 | 2.148 |

**Table S3.** Linear regression fit for *(a)* phenotypic traits per genotype and *(b)* Dimension 1 and Dimension 2 of the FAMD multivariate analysis. Statistical information is shown in columns.


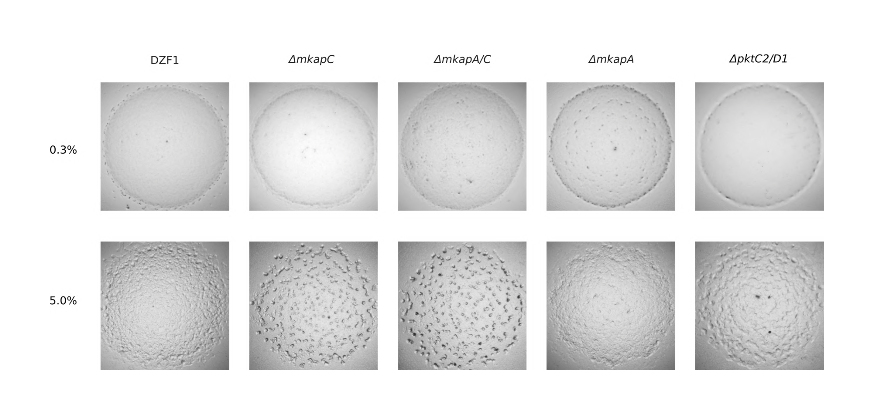


**Figure S4.** Fruiting bodies do not fully develop at 2.5% and 0.3% agar percentages. Note that even if aggregates are visible for some strains, they do not darken, indicating that spores do not differentiate. All micrographs were taken at 96 h.
